# Impact of TriGUARD 3 on cerebral protection in Chinese patients undergoing transcatheter aortic valve replacement

**DOI:** 10.3389/fcvm.2026.1694878

**Published:** 2026-03-18

**Authors:** Yanbin Li, Bin Wang, Shichen Zhou, Yujie Zhou, Mao Chen, Jianfang Luo, Jianan Wang, Jun Jin, Xiaoping Peng, Jianzeng Dong, Zening Jin, Yongjun Wang, Kai Xu, Yaling Han

**Affiliations:** 1 The State Key Laboratory of Frigid Zone Cardiovascular Diseases, Department of Cardiology, General Hospital of Northern Theater Command, Shenyang, Liaoning, China; 2 Department of Cardiology, Benxi Central Hospital, Benxi, Liaoning, China; 3 Department of Cardiology, Beijing Anzhen Hospital, Capital Medical University, Beijing, China; 4 Department of Cardiology, West China Hospital, Sichuan University, Chengdu, Sichuan, China; 5 Department of Cardiology, Guangdong Provincial People’s Hospital, Guangzhou, Guangdong, China; 6 Department of Cardiology, The Second Affiliated Hospital Zhejiang University School of Medicine, Hangzhou, Zhejiang, China; 7 Department of Cardiology, Xinqiao Hospital, Army Medical University, Chongqing, Chongqing, China; 8 Department of Cardiology, The First Affiliated Hospital of Nanchang University, Nanchang, Jiangxi, China; 9 Department of Cardiology, The First Affiliated Hospital of Zhengzhou University, Zhengzhou, Henan, China; 10 Department of Cardiology, Beijing Tiantan Hospital, Capital Medical University, Beijing, China

**Keywords:** bicuspid stenosis, cerebral ischemia, diffusion-weighted imaging, embolic protection, neuroprotection, transcatheter valve replacement

## Abstract

**Introduction:**

In China, evidence regarding cerebral embolic protection device (CEPD) use during transcatheter aortic valve replacement (TAVR) for severe aortic stenosis treatment is limited. This study evaluated the TriGUARD 3 (TG3) CEPD performance in patients undergoing TAVR.

**Methods:**

Data from two studies were pooled: the CEPD group was derived from a multicenter TG3 trial in China, whereas the control group was obtained from a single-center registry. All participants underwent transfemoral TAVR and completed pre- and postoperative diffusion-weighted magnetic resonance imaging (DW-MRI). The primary outcome was total cerebral ischemic lesion volume on DW-MRI.

**Results:**

No significant difference was observed between groups in total lesion volume {CEPD [*n* = 62] vs. control [*n* = 56]; 256.53 [interquartile range (IQR), 44.12–667.99] vs. 271.88 [IQR, 96.10–650.87]; *p* = 0.456}. Median regression analysis in the overall cohort showed no significant association between CEPD use and total lesion volume (*p* = 0.181). Nonetheless, among patients with bicuspid aortic valve (BAV) stenosis, the CEPD group demonstrated significantly lower total lesion volume [165.43 (IQR, 32.96–311.13) vs. 309.38 (IQR, 96.10–788.49); *p* = 0.025], average lesion volume [61.3 (IQR, 23.44–89.65) vs. 93.75 (IQR, 51.73–137.07); *p* = 0.019], and maximum single-lesion volume [89.65 (IQR, 28.13–174.02) vs. 164.14 (IQR, 75.00–365.08); *p* = 0.019]. Median regression revealed that CEPD use was significantly associated with reductions in total, average, and maximum single-lesion volumes (median differences: −406.1, −82.2, and −137.6; all *p* < 0.05), independent of age, sex, hypertension, diabetes, valve type, and pre-dilatation.

**Conclusion:**

In patients with severe aortic stenosis undergoing transfemoral TAVR, TG3 CEPD did not significantly reduce the total lesion volume on DW-MRI. In the BAV subgroup, an association was observed between device use and reductions in total, average, and maximum single-lesion volumes. This exploratory finding is hypothesis-generating and should be further elucidated in larger randomized studies.

## 1 Introduction

Since its introduction in 2002, transcatheter aortic valve replacement (TAVR) has become the treatment of choice for high-surgical-risk patients with severe aortic stenosis (AS). With accumulating clinical evidence and technological advancements, TAVR indications have progressively expanded to include selected intermediate- and low-risk patients (1–6). Despite advancements, stroke remains a common complication, with a 30-day incidence of 2%–6% and a notable mortality burden (7–9). Similarly, periprocedural stroke is associated with a significant increase in mortality rates (10). Moreover, 68%–93% of patients with TAVR exhibit embolic cerebral injuries detectable on postprocedural diffusion-weighted magnetic resonance imaging (DW-MRI) (11–14). Therefore, using cerebral embolic protection devices (CEPD) to shield cerebral arteries from embolic debris during the procedure is critical.

The role of CEPD in TAVR, particularly in reducing the number and volume of ischemic brain lesions on DW-MRI and lowering clinical ischemic stroke risk, remains unclear. While no statistically significant differences were observed in overall clinical endpoints, the PROTECTED TAVR study demonstrated a significant reduction in disabling strokes (14–17).

In addition to the Sentinel device, the TriGUARD Cerebral Protection Device represents another CEPD evaluated in previous randomized controlled trials (RCTs). Although the TriGUARD3 (TG3) CEPD did not confer significant benefits in 30-day clinical outcomes, DW-MRI analyses indicated potential reduction in large-lesion volume (18–20). Nonetheless, TG3 studies were performed exclusively in Western populations, providing limited evidence for Chinese patients. Compared with Western populations, Chinese patients undergoing TAVR exhibit a higher bicuspid aortic valve (BAV) stenosis prevalence (21), which has been associated with more severe cerebral embolization (22). Accordingly, this study evaluated real-world TG3 CEPD effectiveness in Chinese patients undergoing TAVR.

## 2 Materials and methods

### 2.1 Patient population

**Figure 1:**
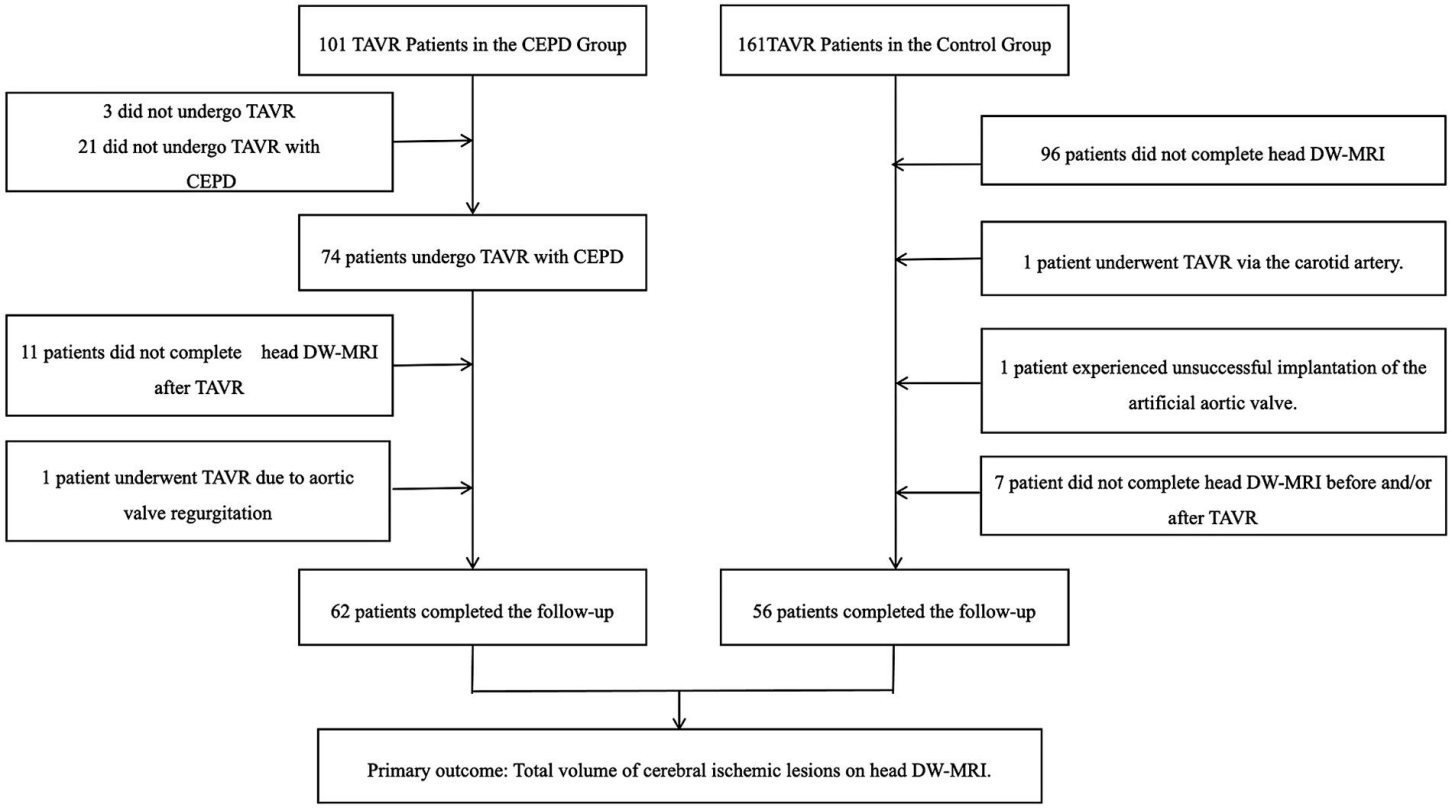
Patient selection flowchart. Patient screening flowchart. Patients were divided into the CEPD and the control groups. All patients underwent head DW-MRI examination before TAVR and within 7 days after TAVR (or until discharge, whichever came first), and all completed a 30-day follow-up. CEPD, cerebral embolic protection device; DW-MRI, diffusion-weighted magnetic resonance imaging; TAVR, transcatheter aortic valve replacement.

Participants were drawn from two studies. The CEPD group was recruited from a prospective, multicenter, single-arm objective performance criteria study conducted from June 2021 to August 2022 (TG3 China Clinical Trial), in which 101 patients with severe aortic stenosis at nine centers in China were screened. Informed consent was obtained from all participants, and the study was approved by the institutional review board at each participating center. Patient safety was overseen by an independent Data and Safety Monitoring Board, and all endpoint events were adjudicated by an independent Clinical Event Committee. Of the screened patients, 74 underwent transfemoral transcatheter aortic valve replacement (TF-TAVR) with TG3 CEPD assistance; 62 completed pre- and postoperative DW-MRI scans, comprising the CEPD group (Supplementary Material 1). The control group was selected from a prospective, single-center registry at the General Hospital of Northern Theater Command, in which 56 patients with severe aortic stenosis who underwent TAVR between December 2021 and July 2023 were enrolled; all completed pre- and postprocedure DW-MRI scans (Figure 1). All participants underwent routine laboratory testing and collection of medical history and current medication data, as well as diagnostic evaluation, including Society of Thoracic Surgeons score, New York Heart Association functional classification, electrocardiography, echocardiography, head DW-MRI, and multidetector computed tomography (analyzed using 3mensio software, Pie Medical Imaging, Maastricht, Netherlands). Inclusion criteria were a confirmed severe aortic stenosis diagnosis, transfemoral TAVR, and completion of pre- and postoperative head DW-MRI scans. Exclusion criteria comprised non-transfemoral TAVR approaches (e.g., transapical, transaxillary, subclavian, transaortic, or transcarotid routes); incomplete pre- and/or post-TAVR head DW-MRI scans; prior aortic valve implantation history; impaired renal function (estimated glomerular filtration rate <30 ml/min) or liver failure; TAVR requirement for aortic regurgitation; and severe aortic arch calcification, significant atherosclerosis, or pronounced arterial tortuosity. TAVR was performed with commercially available transcatheter valve systems according to standard institutional practice. Valves were categorized as self-expanding (VenusA-Valve) or balloon-expandable (Sapien 3). The study adhered to the principles of the Declaration of Helsinki, including participant rights protection, strict patient information confidentiality, and ethics committee approval at all participating centers.

### 2.2 TG3 device

In China, TG3 CEPD is used solely for research purposes and has been granted “green channel” status by the National Medical Products Administration. The TG3 device was deployed before TAVR, remained in place throughout the procedure, and was retrieved afterward. When the TG3 was kept open during all major device-valve interaction steps (including pre-dilation, valve implantation, and post-dilation), the procedure was defined as “complete protection”. When none of these steps involved use of the TG3 system, the procedure was defined as “partial protection”. Detailed information is provided in Supplementary Material 2.

### 2.3 Outcomes and definitions

Primary and secondary outcomes were defined based on the Valve Academic Research Consortium 3 (VARC-3) criteria (23). The primary outcome was total cerebral ischemic lesion volume on head DW-MRI. Secondary outcomes included: 30-day net adverse clinical events (NACE); cerebral ischemic lesion metrics on DW-MRI, including new-lesion incidence, lesion number, average lesion volume, maximum single-lesion volume, and the number of lesions exceeding 600 mm³ and 1,000 mm³; and major and minor TG3 access site-related vascular complications. NACE was defined as a composite of all-cause mortality, disabling and non-disabling strokes, major bleeding (VARC-3 grade or higher), and acute kidney injury (stage 3 or higher). Head DW-MRI identifies diffusion restriction by calculating apparent diffusion coefficient maps. Lesions were defined as focal high-signal areas on fluid-attenuated inversion recovery sequences, corresponding to restricted diffusion on diffusion-weighted imaging; artifacts were excluded by confirmation on apparent diffusion coefficient maps (24). All patients underwent DW-MRI within 30 days before admission and again either before discharge or within 7 days after the procedure. If new lesions were detected on pre-admission DW-MRI, these patients were excluded from the postprocedure DW-MRI analysis. New ischemic lesions on head MRI were analyzed using 3D Slicer software (version 5.7.0), with additional review by a neurologist or radiologist as needed.

### 2.4 Statistical analysis

Data were analyzed using SPSS 26 and R 4.5.2 software. Continuous and categorical variables are presented as means ± standard deviation or median [interquartile range (IQR)] and as frequencies and percentages, respectively. The normality of continuous variables was assessed using histograms and Q-Q plots. Intergroup comparisons used the *t*-test, Mann–Whitney *U*-test, or χ² tests, as appropriate. Lesion volume reproducibility was assessed using intraclass correlation coefficients, with values > 0.75 (*p* < 0.05) indicating good reproducibility for the diagnostic test (Supplementary Material 3). To assess the association between CEPD use and lesion volumes, median (quantile) regression was used as the primary analytical approach, given its robustness to the skewed distribution of the volume data.

## 3 Results

### 3.1 Patient and procedural characteristics

The control and CEPD groups included 56 and 62 patients who underwent TF-TAVR and head DW-MRI and TF-TAVR with TG3 CEPD assistance and head DW-MRI, respectively. Baseline characteristics and differences between the two groups are summarized in Table 1. The mean age (72.18 ± 7.70 vs. 71.79 ± 8.15, respectively; *p* = 0.265), history of atrial fibrillation/flutter (21.4% vs. 22.6%, respectively; *p* = 0.880), and history of carotid artery disease (10.7% vs. 9.7%, respectively; *p* = 0.852) were consistent with a typical TAVR patient population in China. The CEPD group demonstrated a significantly higher pre-implant balloon dilation rate compared to the control group (60.7% vs. 79%, respectively; *p* = 0.030), whereas post-implant balloon dilation rates were similar between groups (32.1% vs. 35.5%, respectively; *p* = 0.702). Self-expanding valve use differed significantly, at 100% in the control group compared to 67.9% in the CEPD group (*p* < 0.001). Baseline demographic characteristics, clinical manifestations, and procedural details in patients with BAV stenosis were analyzed (Supplementary Material 4).

**Table 1:** Baseline demographics, clinical presentation, and procedure details.

Variable	Control group (*N* = 56)	CEPD group (*N* = 62)	*P*-value
Demographics			
Age-year	72.18 ± 7.70	71.79 ± 8.15	0.265
Male-no. (%)	66.1 (37/56)	54.8 (34/62)	0.213
Clinical presentation			
Prior smoking	37.5 (21/56)	32.3 (20/62)	0.550
Hypertension	44.6 (25/56)	48.4 (30/62)	0.684
Diabetes mellitus	28.6 (16/56)	12.9 (8/62)	0.035
LDL-C (mmol/L)	2.32 ± 0.69	2.57 ± 1.25	0.177
Prior atrial fibrillation/atrial flutter	21.4 (12/56)	22.6 (14/62)	0.880
Prior coronary revascularization (CABG or PCI)	14.3 (8/56)	9.7 (6/62)	0.440
Prior stroke or TIA	14.3 (8/56)	4.8 (3/62)	0.078
Stroke more than 6 months prior	10.7 (6/56)	4.8 (3/62)	0.305
Prior renal disease	3.6 (2/56)	3.2 (2/62)	0.917
Prior PVD	39.3 (22/56)	25.8 (16/62)	0.118
Prior aortic disease (aneurysm)	7.1 (4/56)	3.2 (2/62)	0.333
Prior carotid artery disease	10.7 (6/56)	9.7 (6/62)	0.852
NYHA III/IV (%)	51.8 (29/56)	69.4 (43/62)	0.051
mRS score			
0–1	85.7 (48/56)	95.2 (59/62)	0.078
≥2	14.3 (8/56)	4.8 (3/62)	
Monotherapy antiplatelet agents	35.7 (20/56)	43.5 (27/62)	0.385
Dual antiplatelet therapy	25 (14/56)	19.4 (12/62)	0.460
Oral anticoagulants	17.9 (10/56)	24.2 (15/62)	0.400
Calcium score	384 (235.25–842.75)	486 (307.50–773.05)	0.338
STS score	2.10 (1.18–2.86)	2.39 (1.70–3.15)	0.078
Procedure details			
Valve type			
BAV	71.4 (40/56)	56.5 (35/62)	0.091
Balloon pre-dilation	60.7 (34/56)	79 (49/62)	0.030
Balloon post-dilation	32.1 (18/56)	35.5 (22/62)	0.702
Self-expanding valve (VenusA-Valve)	67.9 (38/56)	100 (62/62)	<0.001
Balloon-expandable valve (Sapien 3)	32.1 (18/56)	0 (0/62)	<0.001

Categorical variables are presented as % (*n*/*N*). Continuous variables are presented as means ± standard deviation or median (interquartile range). BAV, bicuspid aortic valve; CABG, coronary artery bypass grafting; CEPD, cerebral embolic protection device; NYHA, New York Heart Association; PCI, percutaneous coronary intervention; PVD, peripheral vascular disease; STS, Society of Thoracic Surgeons; TIA, transient ischemic attack.

### 3.2 DW-MRI results in overall patients

All participants underwent head DW-MRI before and after the procedure. The postprocedural DW-MRI results demonstrated no statistically significant difference in the total lesion volume between the TG3 CEPD and the control groups [256.53 (IQR, 44.12–667.99) vs. 271.88 (IQR, 96.10–650.87), respectively; *p* = 0.456]. Further analysis revealed no significant differences between the groups in new lesion incidence, lesion number, maximum lesion volume, or the number of lesions exceeding 600 mm³ or 1,000 mm³. Nevertheless, the average lesion volume tended to be lower in the CEPD group (Table 2). Notably, when comparing the fully covered CEPD subgroup with the control group, the trend toward a reduced average lesion volume was more pronounced (Supplementary Material 5). Median regression analysis in the overall cohort further indicated no significant association between CEPD use and total lesion volume (*p* = 0.181) (Supplementary Material 6).

**Table 2:** Brain lesion characteristics as determined by magnetic resonance imaging.

Variable	Control group (*N* = 56)	CEPD group (*N* = 62)	*P*-value
Primary outcome			
Total lesion volume	271.88 (96.10–650.87)	256.53 (44.12–667.99)	0.456
Secondary outcome			
Average lesion volume	90.63 (51.56–117.75)	69.89 (38.77–102.64)	0.096
Maximum volume of a single lesion	131.25 (75.00–276.56)	106.71 (38.77–215.46)	0.190
New lesion rate	87.5 (49/56)	82.3 (51/62)	0.429
Number of lesions	3 (1–6)	3 (1–7)	0.903
Lesions larger than 600 mm³	16.1 (9/56)	9.7 (6/62)	0.298
Lesions larger than 1,000 mm³	7.1 (4/56)	1.6 (1/62)	0.189

Categorical and continuous variables are presented as % (*n*/*N*) and medians (interquartile range).

### 3.3 DW-MRI results in BAV patients

**Figure 2:**
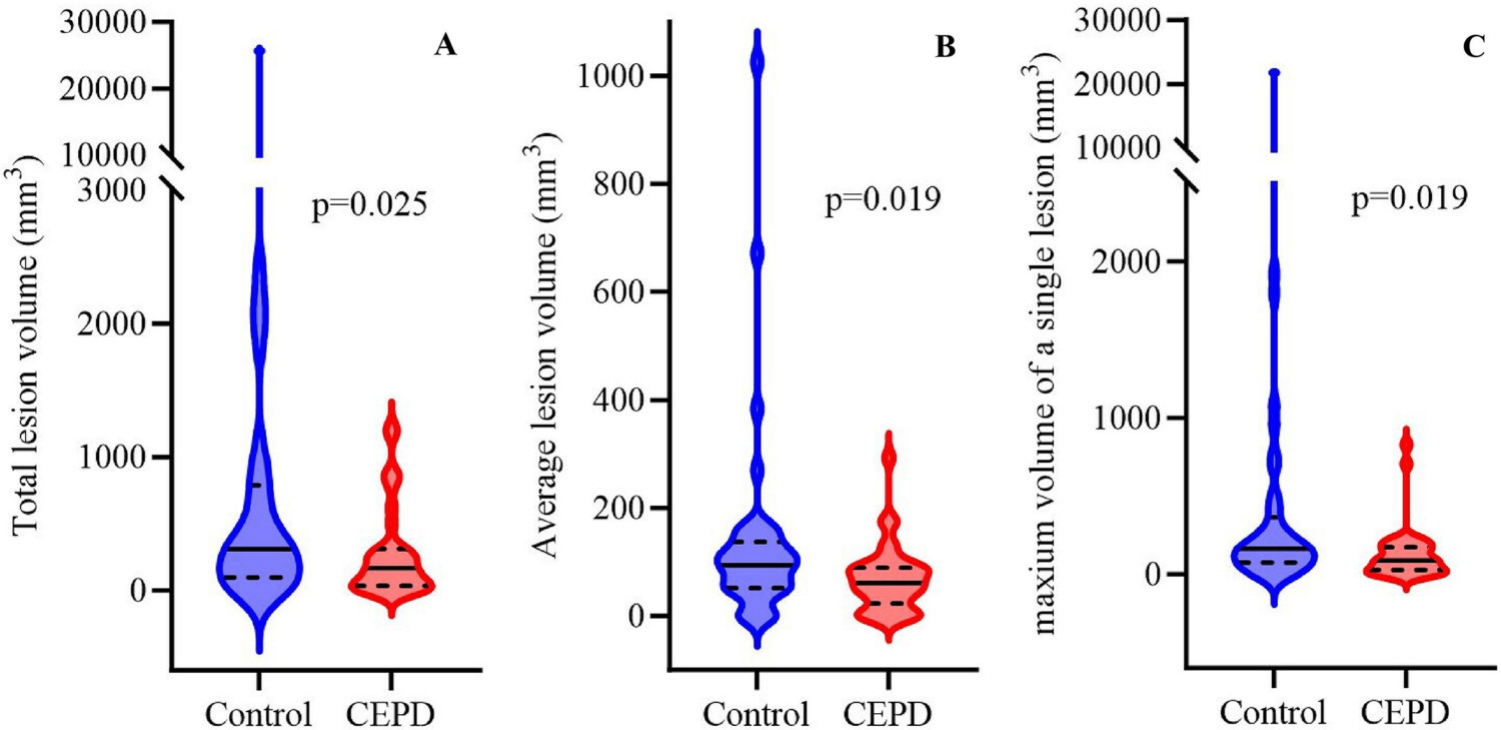
Head DW-MRI (BAV). Patients with bicuspid aortic valve stenosis were divided into a control group and a CEPD group. Head DW-MRI findings were summarized using the following ischemic lesion volume indices: **(A)** total lesion volume, **(B)** average lesion volume, and **(C)** maximum volume of the individual lesion. DW-MRI, diffusion-weighted magnetic resonance imaging; BAV, bicuspid aortic valve.

In the BAV subgroup, statistically significant differences were observed between the CEPD (*n* = 35) and control (*n* = 40) groups. The total lesion volume in the CEPD group [165.43 (IQR, 32.96–311.13)] was significantly lower than that in the control group [309.38 (IQR, 96.10–788.49), *p* = 0.025]. Further analysis revealed that both the average lesion volume [61.3 (IQR, 23.44–89.65)] and the maximum single-lesion volume [89.65 (IQR, 28.13–174.02)] in the CEPD group were significantly lower than those in the control group [93.75 (IQR, 51.73–137.07) and 164.14 (IQR, 75.00–365.08), respectively; both *p* = 0.019]. No statistically significant differences were found between the two groups regarding the incidence of new lesions (80% vs. 87.5%, respectively; *p* = 0.377), the number of lesions [2 (IQR, 1–4) vs. 3 (IQR, 1–7), respectively; *p* = 0.139], and the proportion of lesions exceeding thresholds of 600 mm³ (5.7% vs. 17.5%, respectively; *p* = 0.162) and 1,000 mm³ (0% vs. 10%, respectively; *p* = 0.118) (Figure 2 and Supplementary Material 7). Median regression analyses revealed that CEPD use was significantly associated with lower lesion volumes. Specifically, CEPD use was associated with reduced total lesion volume (median difference = −406.1, 95% CI: −802.9 to −9.4, *p* = 0.049), average lesion volume (median difference = −82.2, 95% CI: −137.6 to −26.8, *p* = 0.005), and maximum single lesion volume (median difference = −137.6, 95% CI: −263.4 to −11.9, *p* = 0.036). These associations remained statistically significant after adjustment for age, sex, hypertension, diabetes mellitus, valve type, and pre-dilatation, none of which were significant predictors (Table 3, Supplementary Materials 8, 9).

**Table 3:** Median regression assessing the association between CEPD and total lesion volume in BAV patients.

Variable	Coefficient	95% CI	*P*-value
CEPD usage	−406.1	(−802.9, −9.4)	0.049
Age	−3.5	(−30.7, 23.8)	0.802
Female	285.8	(−118.4, 690.1)	0.170
Hypertension	−33.8	(−344.6, 277.0)	0.832
Diabetes mellitus	−33.8	(−638.0, 570.4)	0.913
Valve type (Self-expanding valve)	219.1	(−271.0, 709.2)	0.384
Pre-dilatation	−49.1	(−366.4, 268.3)	0.763

CEPD, cerebral embolic protection device; CI, confidence interval.

Further analyses of aortic valve morphology and brain lesion characteristics revealed no statistically significant between-group differences (CEPD vs. control) among patients with tricuspid aortic valve in the incidence of new lesions, lesion number, average lesion volume, maximum single-lesion volume, or the proportions of lesions exceeding 600 mm³ and 1,000 mm³ (Supplementary Material 10). When the CEPD group was subdivided into BAV and TAV subgroups, the former subgroup demonstrated significantly lower total lesion volume and fewer new lesions compared to the latter (Supplementary Material 11). Furthermore, among patients in the control group, when divided into BAV and TAV subgroups, no statistically significant differences were observed in parameters such as total lesion volume and average lesion volume (Supplementary Material 12).

### 3.4 Clinical outcomes

The 30-day follow-up rates for both groups were 100%. During the 30-day clinical evaluation post-TAVR (Table 4), the incidence of the secondary outcome NACE was comparable between the CEPD and the control groups (8.1% vs. 8.9%, respectively; *p* = 1.000). Neither group experienced a disabling stroke or severe complications, including stage 3 or higher acute kidney injury. During follow-up, two deaths occurred in the CEPD group: one of unknown cause after the patient presented with respiratory difficulty and fever, and one of cardiogenic cause associated with atrioventricular block. In the control group, one death occurred 15 days after discharge, also of unknown cause. Stroke incidence was lower in the CEPD group than in the control group (1.6% vs. 3.6%), but the difference was not statistically significant (*p* = 0.603). Detailed MRI data for the stroke cases in this cohort are presented in Supplementary Material 13. Major bleeding complications were slightly more frequent in the CEPD group (3.2%) than in the control group (1.8%), but the difference was not statistically significant (*p* = 1.000). No major vascular complications were observed in either group. Nonetheless, minor vascular complications occurred at similar (non-significantly different) rates, with two TAVR access-related events in each group (3.2% vs. 3.6%, respectively; *p* = 1.000). In the CEPD group, two minor vascular complications were explicitly related to TG3 access: femoral artery occlusion (resolved with balloon dilation) and femoral access site bleeding (managed with compression and transfusion). Both complications were treated promptly and effectively. In the BAV subgroup analysis, no statistically significant between-group differences in clinical outcomes were observed when comparing CEPD vs. control (Supplementary Material 7).

**Table 4:** Clinical outcomes.

Variable	Control group (*N* = 56)	CEPD group (*N* = 62)	*P*-value
NACE	8.9 (5/56)	8.1 (5/62)	1.000
All-cause mortality	1.8 (1/56)	3.2 (2/62)	1.000
Stroke	5.4 (3/56)	1.6 (1/62)	0.345
Disabling stroke	0.00 (0/56)	0.00 (0/62)	NA
Non-disabling stroke	5.4 (3/56)	1.6 (1/62)	0.345
Acute kidney failure (stage 3 or higher)	0.00 (0/56)	0.00 (0/62)	NA
Life-threatening bleeding (VARC type 3 or higher)	1.8 (1/56)	3.2 (2/62)	1.00
Vascular complications related to TG3			
Major vascular complications	0.00 (0/56)	0.00 (0/62)	NA
Minor vascular complications	0.00 (0/56)	3.2 (2/62)	0.497
Vascular complications related to the TAVR			
Major vascular complications	0.00 (0/56)	0.00 (0/62)	NA
Minor vascular complications	3.6 (2/56)	3.2 (2/62)	1.000

Categorical variables are presented as % (*n*/*N*), NACE, net adverse clinical events; TAVR, transcatheter aortic valve replacement; TG3, TriGUARD 3.

### 3.5 TG3 performance

Overall, 62 patients with severe AS underwent TF-TAVR with TG3 CEPD support. All procedures used a single TG3 device, which was deployed and retrieved smoothly via the contralateral femoral artery. Complete coverage of the three cerebral vessels was achieved in 58 patients, whereas four patients had partial coverage. In three cases, full protection of the three cerebral vessels was achieved before valve deployment; nonetheless, after valve release, the TG3 device shifted and covered only two vessels. In the fourth case, interference between the valve delivery system and the TG3 system prevented accurate positioning of the valve delivery system, requiring early withdrawal of the TG3 system before valve release (Supplementary Material 14).

## 4 Discussion

This study is the first in a Chinese population to evaluate TG3 CEPD efficacy during TF-TAVR. Comparison of DW-MRI data between the CEPD and control groups demonstrated no significant differences in total lesion volume, average lesion volume, new ischemic lesion incidence, lesion number, maximum lesion volume, or lesion volumes exceeding 600 mm³ or 1,000 mm³. Nonetheless, subgroup analysis of patients with BAV exhibited significant reductions in total lesion volume, mean lesion volume, and maximum single-lesion volume with TG3 use (median differences: −406.1, −82.2, and −137.6, respectively; all *p* < 0.05). At the 30-day follow-up, no significant between-group differences were observed in NACE incidence, disabling stroke, major vascular complications, or stage 3 or higher acute kidney injury. Additionally, the non-disabling stroke incidence did not differ significantly between the CEPD and control groups (1.6% vs. 5.4%, respectively; *p* = 0.345). Mortality rates were also similar between groups.

Stroke is a severe TAVR complication; specifically, perioperative stroke increases 30-day mortality risk by more than sixfold (25). Perioperative strokes are mainly attributable to embolic events during the procedure, whereas late strokes may reflect device-related factors or spontaneous causes. Emboli may originate from calcified native aortic valves, atherosclerotic plaques in the aorta, thrombi, and other sources (26), and these events commonly occur during balloon pre-dilation, balloon post-dilation, valve implantation, and catheter manipulation (27, 28). In a follow-up study of 512 patients, stroke following TAVR was associated with longer intensive care unit and hospital stays, higher in-hospital mortality, and increased 30-day cardiovascular mortality (29). Furthermore, the ASTRO-TAVR study indicated that perioperative strokes typically occur within 0–2 days following the procedure and are associated with high mortality. Stroke severity is positively correlated with mortality, and use of neurointerventional therapies, such as thrombolytic drugs or mechanical thrombectomy, remains low. Nonetheless, timely intervention improves survival, emphasizing the need for early management of perioperative stroke (30). The PROTECTED TAVR RCT of CEPD use during TAVR included 3,000 patients with aortic stenosis. Patients were randomized 1:1 to the SENTINEL CEPD group or the control group. The primary endpoint, perioperative stroke incidence, did not differ significantly between the CEPD and control groups, whereas disabling stroke incidence was lower in the CEPD group. No significant differences were observed in non-disabling stroke, all-cause mortality, stroke plus transient ischemic attack, or acute kidney injury between groups (17). Our findings align with those of the PROTECTED TAVR trial; nonetheless, no disabling strokes occurred in either group, likely because of the relatively small sample size. Unlike the SENTINEL CEPD, which does not provide coverage of the left vertebral artery, the TG3 CEPD used here provided complete coverage of the three major cerebral vessels, including the left vertebral artery. To assess the potential impact of this design difference, we compared new ischemic lesion incidence in the left vertebral artery between the control and TG3 CEPD groups. New ischemic lesions in the left vertebral artery occurred in 5.4% (3/56) of patients in the control group and in 0% (0/62) of patients in the TG3 CEPD group. Although the difference was not statistically significant (*p* = 0.104), it suggested a trend toward lower new lesion incidence in the left vertebral artery with TG3 CEPD use. This observation supports a potential advantage of TG3 CEPD in preventing TAVR-related cerebrovascular events and warrants further investigation and validation in future studies.

Our findings are consistent with those of the REFLECT II study, which demonstrated successful TG3 deployment and retrieval in the aortic arch. The TG3 coverage rate in our study (93.5% during pre-, intra-, and post-TAVR) exceeded the previously reported 59.7%, likely reflecting rigorous preprocedural screening that excluded patients with severe aortic arch calcification, significant atherosclerosis, or highly tortuous vessels. Major TG3-related vascular complications were not observed here, compared with a 1.9% rate in REFLECT II, supporting TG3 safety and efficacy. Two minor complications occurred in the CEPD group: femoral artery access-site occlusion, resolved by balloon dilation, and access-site bleeding controlled with compression and transfusion; both events were managed promptly without long-term effects. In REFLECT II, median total lesion volumes were comparable between the CEPD and control groups (215 mm³ vs. 188 mm³, *p* = 0.405). Similarly, we found no significant between-group difference in median total lesion volume between the TG3 and control groups (271.88 mm³ vs. 256.53 mm³, *p* = 0.456), in line with prior results. However, compared with REFLECT II, total lesion volume, average lesion volume, and maximum single-lesion volume were larger in our study. This difference may be attributable to the higher BAV proportion in our cohort (56.5% in the CEPD group and 71.4% in the control group).

The TORCH study confirmed that patients with BAV undergoing TAVR develop more severe ischemic brain lesions, with higher lesion numbers and larger lesion volumes, compared to patients with TAV. In our control group, patients were subdivided into TAV (*n* = 16) and BAV (*n* = 40) subgroups. Median lesion number, total lesion volume, average lesion volume, and maximum single-lesion volume indicated more severe head DW-MRI lesions in patients with BAV. Further DW-MRI analysis in patients with BAV, comparing the control group (*n* = 40) with the CEPD group (*n* = 35), demonstrated significantly lower median total lesion volume, average lesion volume, and maximum single-lesion volume in the CEPD group (*p* = 0.025, *p* = 0.019, and *p* = 0.019, respectively). Median regression further revealed that CEPD use was significantly associated with reductions in total, average, and maximum single-lesion volumes (median differences: −406.1, −82.2, and −137.6; all *p* < 0.05), independent of age, sex, hypertension, diabetes, valve type, and pre-dilatation. Collectively, these findings suggest a greater CEPD benefit in patients with BAV, reflected by lower lesion volumes. The prospective, multicenter, randomized BHF PROTECT-TAVR trial aims to enroll 7,635 patients randomized 1:1 to TAVR with or without CEPD. In that trial, CEPD use did not reduce stroke incidence within 72 h, patients with BAV accounted for only 8.4% of the cohort, and no head DW-MRI analysis was performed (31). Conversely, our population comprised a substantially higher BAV proportion (63.6%) and included head DW-MRI analysis. Because BAV is associated with earlier valve calcification and stenosis, severe aortic stenosis occurs earlier. CEPD use during TAVR in BAV inpatients may provide clinical benefits for the following reasons: (1) BAV is generally more prone to severe calcification; during TAVR, calcified valve fragments may dislodge into cerebral vessels, increasing embolism risk. (2) BAV is often associated with complex anatomical variation, including irregular annular shape and abnormal aortic root morphology, which increases TAVR complexity and the likelihood of debris dislodgment.

Patients with BAV stenosis are typically younger than those with TAV stenosis and have a longer life expectancy, making postoperative strokes particularly impactful for this population. Few clinical trials have evaluated the benefits of CEPD based on DW-MRI findings in patients with severe BAV stenosis undergoing TF-TAVR. Our study demonstrated the benefits of CEPD in TF-TAVR for patients with severe BAV stenosis on DW-MRI, providing a promising foundation for the design and analysis of future clinical research.

### 4.1 Limitations

This exploratory study had certain limitations. First, the retrospective design, with a multicenter experimental group and a single-center control group, introduces inherent selection bias that could not be fully mitigated. Second, the relatively small sample size and the single-center control group warrant larger, multicenter randomized controlled trials to validate TG3 efficacy. Propensity score matching was not performed to preserve the representativeness of TG3 use in the Chinese population, as matching can further reduce sample size. Additionally, this study used an imaging biomarker (DW-MRI lesion volume) as the primary endpoint rather than direct clinical measures of stroke or neurocognitive function. Although prior evidence supports an association between these lesions and adverse neurological outcomes, the absence of systematic neuropsychological assessment precludes definitive conclusions regarding clinical impact. Therefore, the clinical relevance of reduced lesion volumes remains uncertain and requires confirmation in future studies incorporating dedicated neurocognitive endpoints.

## 5 Conclusion

In Chinese patients with severe aortic stenosis undergoing TF-TAVR, use of a TG3 cerebral protection device did not reduce the total lesion volume observed on head DW-MRI. In the BAV subgroup, an association was observed between device use and reductions in total, average, and maximum single-lesion volumes. This exploratory finding is hypothesis-generating and should be further elucidated in larger randomized studies.

## Data Availability

The datasets presented in this article are not readily available due to restrictions that include that it is intended for academic review and research-related verification within the scope of this submission. Any further use, such as secondary analysis, redistribution, or modification, requires prior consultation with the corresponding author(s). Requests to access the datasets should be directed to Kai Xu, xukai2001@sina.com.

## Supplementary material

The Supplementary Materialfor this article can be found online at: https://www.frontiersin.org/articles/10.3389/fcvm.2026.1694878/full#supplementary-material
