# Supplementary material for: Impact of TriGUARD 3 on cerebral protection in Chinese patients undergoing transcatheter aortic valve replacement
Source: Front Cardiovasc Med. 2026 Mar 18;13:1694878. doi: 10.3389/fcvm.2026.1694878 (PMC13038980; doi:10.3389/fcvm.2026.1694878)
Supplement: Supplementary file 1 [file Datasheet1.docx]

Supplementary Material

# Supplementary Material 1

**Distribution of Patients Among Nine Centers in China**

| **The names of 9 centers in China** | **CEPD group（n=62）** | **Control group（n=56）** |
| --- | --- | --- |
| General Hospital of Northern Theater Command | 23 | 56 |
| Beijing Tiantan Hospital, Capital Medical University | 1 | 0 |
| Beijing Anzhen Hospital, Capital Medical University | 13 | 0 |
| West China Hospital, Sichuan University | 6 | 0 |
| The Second Affiliated Hospital, Zhejiang University School of Medicine | 5 | 0 |
| Xinqiao Hospital, Army Medical University | 3 | 0 |
| The First Affiliated Hospital of Zhengzhou University | 2 | 0 |
| Guangdong Provincial People's Hospital | 6 | 0 |
| The First Affiliated Hospital of Nanchang University | 3 | 0 |

CEPD, cerebral embolic protection device.

# Supplementary Material 2.

**Detailed Information of The TriGUARD 3™.**

The TriGUARD 3™ is a temporary, retrievable, single-use aortic embolic deflection device designed to cover the three major cerebral artery branches originating from the aortic arch (the brachiocephalic artery, left common carotid artery, and left subclavian artery). Its primary objective is to reduce embolic material entry into the cerebral circulation during transcatheter cardiac procedures, such as TAVR.

The device consists of a self-expanding radiopaque Nitinol frame integrated with a biocompatible polymer mesh filter (nominal pore size, 115 × 145 μm). It is advanced into the aortic arch through an 8-F femoral artery sheath under fluoroscopic guidance. Notably, the sheath can simultaneously accommodate a pigtail catheter, eliminating the need for an additional arterial access site. After deployment, the device expands to cover the ostia of the target cerebral arteries and is anchored through circumferential frame apposition against the aortic wall. An integrated lumen permits pigtail catheter placement for aortic root angiography during the procedure.

When positioned, the device diverts embolic debris away from the cerebral circulation into the descending aorta, where such material is less likely to cause injury and can be safely metabolized, while maintaining uninterrupted cerebral blood flow. The intended procedural sequence involves device deployment before TAVR, maintenance of its position throughout the TAVR procedure, and retrieval after procedure completion. The procedure is defined as achieving “complete protection” when the device remains fully functional throughout all critical TAVR steps (pre-dilation, valve deployment, and post-dilation).

Regarding regulatory status, TriGUARD 3™ has obtained CE Mark approval in Europe. In China, the device is currently approved for investigational use only and has been granted “Green Channel” status by the National Medical Products Administration (NMPA). For comprehensive operational details, please refer to the TriGUARD 3™ Instructions for Use.

**
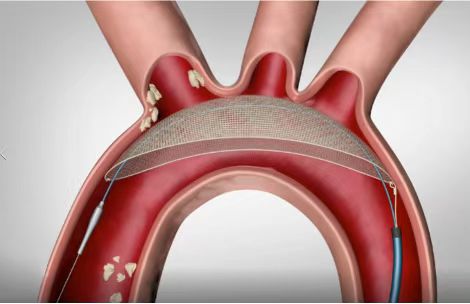
**

# Supplementary Material 3

**Reproducibility assessment：**

A random sample of 10 study subjects was selected to assess the reproducibility of cranial ischemic lesion parameters measured using diffusion-weighted magnetic resonance imaging (DW-MRI) using a 3D Slicer. For interobserver reproducibility, two independent operators measured and analyzed DW-MRI ischemic lesion parameters. For intraobserver reproducibility, each operator performed at least two separate parameter measurements. Interobserver and intraobserver reproducibility were evaluated using intraclass correlation coefficients (ICC). An ICC value >0.75 (p<0.05) was considered indicative of good reproducibility for the diagnostic test (see table below).

**Evaluation of Interobserver and Intraobserver Reproducibility of Measured Parameters**

| **Parameters** | **Interobserver** | | |  | **Intraobserver** | | |
| --- | --- | --- | --- | --- | --- | --- | --- |
|  | **ICC** | **95%CI** | **P-value** |  | **ICC** | **95%CI** | **P-value** |
| total lesion volume | 0.980 | 0.912–0.995 | ＜0.001 |  | 0.975 | 0.870–0.994 | ＜0.001 |
| average lesion volume | 0.978 | 0.906–0.995 | ＜0.001 |  | 0.951 | 0.728–0.989 | ＜0.001 |
| maximum volume of a single lesion | 0.979 | 0.917–0.996 | ＜0.001 |  | 0.972 | 0.887–0.993 | ＜0.001 |

3D, three-dimensional; CI, confidence interval; DW-MRI, diffusion-weighted magnetic resonance imaging; ICC, intraclass correlation coefficient.

# Supplementary Material 4

**Baseline Demographics, Clinical Presentation, and Procedure Details (BAV)**

|  | **Control group（N=40）** | **CEPD group（N=35）** | **P-value** |
| --- | --- | --- | --- |
| **Demographics** |  |  |  |
| Age-yr | 71.18 ± 6.81 | 71.69 ± 8.00 | 0.766 |
| Male-no.(%) | 62.5（25/40） | 54.3（19/35） | 0.471 |
| **Clinical Presentation** |  |  |  |
| Prior smoking | 37.5（15/40） | 40.0（14/35） | 0.824 |
| Hypertension | 37.5（15/40） | 42.9（15/35） | 0.637 |
| Diabetes mellitus | 22.5（9/40） | 20.0（7/35） | 0.792 |
| LDL-C (mmol/L)） | 2.29 ± 0.60 | 2.40 ± 0.85 | 0.505 |
| Prior atrial fibrillation/atrial  flutter | 17.5（7/40） | 20.0（7/35） | 0.880 |
| Prior coronary revascularization (CABG or PCI) | 10.0（4/40） | 5.7（2/35） | 0.679 |
| Prior stroke or TIA | 12.5（5/56） | 2.9（1/62） | 0.206 |
| Prior renal disease | 5.0（2/40） | 5.7（2/35） | 1.000 |
| Prior PVD | 35（14/40） | 31.4（11/35） | 0.743 |
| Prior aortic disease  (aneurysm) | 5.0（2/40） | 2.9（1/35） | 1.000 |
| Prior carotid artery disease | 7.5（3/40） | 5.7（2/35） | 1.000 |
| NYHA III/IV（%） | 50.0（20/40） | 68.6（24/35） | 0.103 |
| mRS score |  |  | 0.355 |
| 0–1 | 85.0（34/40） | 94.3（33/35） |  |
| ≥ 2 | 15.0（6/40） | 5.7（2/35） |  |
| Monotherapy antiplatelet agents | 35.0（14/40） | 42.9（15/35） | 0.486 |
| Dual antiplatelet therapy | 25.0（10/40） | 20.0（7/35） | 0.606 |
| Oral anticoagulants | 10.0（4/40） | 14.3（5/35） | 0.831 |
| Calcium Score | 537.5 (276.3, 945.0) | 555.5 (327.0, 844.3) | 0.862 |
| STS score | 1.55（1.09–2.44） | 2.42（1.93–3.12） | 0.004 |
| **Procedure Details** |  |  |  |
| Valve type |  |  |  |
| Balloon pre-dilation | 80.0（32/40） | 77.1（27/35） | 0.763 |
| Balloon post-dilation | 37.5（15/40） | 37.1（13/35） | 0.975 |
| Self-expanding valve (VenusA-Valve) | 65.0（26/40） | 100 (35/35) | < 0.001 |
| Balloon-expandable valve (Sapien 3) | 35.0（14/40） | 0 (0/35) | < 0.001 |

Categorical variables are presented as %(n/N); Continuous variables are presented as means ± standard deviation or median (interquartile range). BAV, bicuspid aortic valve; CABG, coronary artery bypass grafting; CEPD, cerebral embolic protection device; LDL-C, low-density lipoprotein cholesterol; mRS, modified Rankin Scale; N, number (sample size); NYHA, New York Heart Association; PCI, percutaneous coronary intervention; PVD, peripheral vascular disease; STS, Society of Thoracic Surgeons; TIA, transient ischemic attack.

# Supplementary Material 5

# Brain Lesion Characteristics as Determined by Magnetic Resonance Imaging（TG3 complete coverage）

|  | **Control group（N=56）** | **CEPD group（N=58）** | **P-value** |
| --- | --- | --- | --- |
| Primary Endpoint |  |  |  |
| Total lesion volume | 271.88（96.10–650.87） | 216.54（38.77–572.17） | 0.235 |
| Secondary endpoint |  |  |  |
| Average lesion volume | 90.63（51.56–117.75） | 67.73（35.92– 102.64） | 0.076 |
| Maximum volume of a single lesion | 131.25（75.00–276.56） | 102.77（35.92–207.61） | 0.130 |
| New lesion rate | 87.5（49/56） | 81(47/58) | 0.344 |
| Number of lesions | 3（1–6） | 3（1–4.25） | 0.528 |
| Lesions larger than 600 mm³ | 16.1（9/56） | 8.6(5/58) | 0.226 |
| Lesions larger than 1000 mm³ | 7.1（4/56） | 1.7(1/58) | 0.202 |

Categorical variables are presented as %(n/N); Continuous variables are presented as median (interquartile range). CEPD, cerebral embolic protection device.

# Supplementary Material 6

**Median regression assessing the association between CEPD and total lesion volume** **in all patients.**

| **Variable** | **Coefficient** | **95%CI** | | **P-value** |  |
| --- | --- | --- | --- | --- | --- |
| CEPD Usage | -182.8 | (-448.8, -83.3) | 0.181 | | |
| Age | -0.9 | (-13.9, 12.1) | 0.889 | | |
| Female | 136.5 | (-129.7, 402.7) | 0.317 | | |
| Hypertension | 23.0 | (-204.3, 250.3) | 0.843 | | |
| Diabetes mellitus | 27.3 | (-388.9, 443.5) | 0.898 | | |
| Valve type (Self-expanding valve) | 49.7 | (-298.3, 397.6) | 0.780 | | |
| Pre-dilatation | -56.7 | (-288.4, 175.1) | 0.633 | | |

CEPD, cerebral embolic protection device; CI, confidence interval.

# Supplementary Material 7

# Cerebral DW-MRI AND Clinical Outcomes in BAV patients

|  | Control group（N=40） | CEPD group（N=35） | P-value | |
| --- | --- | --- | --- | --- |
| Cerebral DW-MRI |  |  | |  |
| New lesion rate | 87.5 (35/40) | 80 (30/35) | | 0.377 |
| Number of lesions | 3（1–7） | 2（1–4） | | 0.139 |
| Total lesion volume | 309.38 (96.10–788.49) | 165.43 (32.96–311.13) | | 0.025 |
| Average lesion volume | 93.75 (51.73–137.07) | 61.3 (IQR, 23.44–89.65) | | 0.019 |
| Lesions larger than 600 mm³ | 17.5 (7/40) | 5.7 (2/35) | | 0.162 |
| Lesions larger than 1000 mm³ | 10 (4/40) | 0 (0/35) | | 0.118 |
| Maximum volume of a single lesion | 164.14 (75.00–365.08) | 89.65 (28.13–174.02) | | 0.019 |
| Clinical outcomes |  |  | |  |
| NACE | 7.5（3/40） | 5.7（2/35） | | 1.000 |
| All-cause mortality | 0（0/40） | 2.9（1/35） | | 0.467 |
| Stroke | 5.0（2/40） | 0（0/35） | | 0.495 |
| Disabling stroke | 0（0/40） | 0（0/35） | | NA |
| Non-disabling stroke | 5.0（2/40） | 0（0/35） | | 0.495 |
| Acute kidney failure (stage 3 or higher) | 0（0/40） | 0（0/35） | | NA |
| Life-threatening bleeding (VARC type 3 or higher) | 2.5（1/40） | 2.9（1/35） | | 1.000 |

Categorical variables are presented as %(n/N); Continuous variables are presented as median (interquartile range). BAV, bicuspid aortic valve; CEPD, cerebral embolic protection device; DW-MRI, diffusion-weighted magnetic resonance imaging; IQR, interquartile range; NA, not applicable; NACE, net adverse clinical events; VARC, Valve Academic Research Consortium.

# Supplementary Material 8

**Median regression assessing the association between CEPD and average lesion volume in BAV patients.**

| **Variable** | **Coefficient** | **95%CI** | | **P-value** |  |
| --- | --- | --- | --- | --- | --- |
| CEPD Usage | -82.2 | (-137.6, -26.8) | 0.005 | | |
| Age | 0.1 | (-2.9, 3.1) | 0.954 | | |
| Female | 34.0 | (-20.5, 88.5) | 0.226 | | |
| Hypertension | -19.1 | (-57.6, 19.5) | 0.336 | | |
| Diabetes mellitus | -18.4 | (-86.5, 49.8) | 0.599 | | |
| Valve type (Self-expanding valve) | -10.1 | (-87.5, 67.2) | 0.798 | | |
| Pre-dilatation | -12.8 | (-58.7, 33.1) | 0.586 | | |

CEPD, cerebral embolic protection device; CI, confidence interval.

# Supplementary Material 9

**Median regression assessing the association between CEPD and maximum single lesion volume in BAV patients.**

| **Variable** | **Coefficient** | **95%CI** | | **P-value** |  |
| --- | --- | --- | --- | --- | --- |
| CEPD Usage | -137.6 | (-263.4, -11.9) | 0.036 | | |
| Age | -0.7 | (-8.5, 7.0) | 0.850 | | |
| Female | 74.0 | (-34.0, 182.0) | 0.184 | | |
| Hypertension | -19.6 | (-109.2, 69.9) | 0.669 | | |
| Diabetes mellitus | -41.5 | (-267.0, 184.1) | 0.720 | | |
| Valve type (Self-expanding valve) | -3.4 | (-136.0, 129.3) | 0.960 | | |
| Pre-dilatation | -30.9 | (-127.2, 65.3) | 0.531 | | |

CEPD, cerebral embolic protection device; CI, confidence interval.

# Supplementary Material 10

# Cerebral DW-MRI in TAV patients

|  | Control group（N=16） | CEPD group（N=27） | P-value |
| --- | --- | --- | --- |
| Total lesion volume | 227.48（96.10–328.83） | 473.29（50.10–1033.59） | 0.182 |
| Average lesion volume | 81.29（46.87–105.47） | 89.60（50.10–130.71） | 0.392 |
| Maximum volume of a single lesion | 93.75（67.97–201.27） | 147.55（50.10–351.00） | 0.473 |
| New lesion rate | 87.5 (14/16) | 85.2 (23/27) | 1.000 |
| Number of lesions | 3（1.25–3.75） | 4（1–12） | 0.129 |
| Lesions larger than 600 mm³ | 12.5 (2/16) | 14.8 (4/27) | 1.000 |
| Lesions larger than 1000 mm³ | 0 (0/16) | 3.7 (1/27) | 1.000 |

Categorical variables are presented as %(n/N); Continuous variables are presented as median (interquartile range). CEPD, cerebral embolic protection device; DW-MRI, diffusion-weighted magnetic resonance imaging; TAV, tricuspid aortic valve.

# Supplementary Material 11

# Cerebral DW-MRI (CEPD Group)

|  | TAV（N=27） | BAV（N=35） | P-value |
| --- | --- | --- | --- |
| New lesion rate | 85.2（23/27） | 80（28/35） | 0.742 |
| Number of lesions | 4（1–12） | 2（1–4） | 0.029 |
| Total lesion volume | 473.29（50.10–1033.59） | 165.43（32.96–311.13） | 0.016 |
| Average lesion volume | 89.60（50.10–130.71） | 61.30（23.44–89.65） | 0.122 |
| Lesions larger than 600 mm³ | 14.8（4/27） | 5.7（2/35） | 0.390 |
| Lesions larger than 1000 mm³ | 3.7（1/27） | 0（0/35） | 0.435 |
| Maximum volume of a single lesion | 147.55（50.10–351.00） | 89.65（28.13–174.02） | 0.063 |

Categorical variables are presented as %(n/N); Continuous variables are presented as median (interquartile range). BAV, bicuspid aortic valve; CEPD, cerebral embolic protection device; DW-MRI, diffusion-weighted magnetic resonance imaging; TAV, tricuspid aortic valve.

# Supplementary Material 12

# Cerebral DW-MRI (Control Group)

|  | TAV（N=16） | BAV（N=40） | P-value |
| --- | --- | --- | --- |
| New lesion rate | 87.5(14/16) | 87.5(35/40) | 1.000 |
| Number of lesions | 3（1.25–3.75） | 3（1–7） | 0.476 |
| Total lesion volume | 227.48（96.10–328.83） | 309.38（96.10–788.49） | 0.288 |
| Average lesion volume | 81.29（46.87–105.47） | 93.75（51.73–137.07） | 0.197 |
| Lesions larger than 600 mm³ | 12.5(2/16) | 17.5(7/40) | 0.954 |
| Lesions larger than 1000 mm³ | 0(0/16) | 10(4/40) | 0.315 |
| Maximum volume of a single lesion | 93.75（67.97–201.27） | 164.14（75–365.08） | 0.179 |

Categorical variables are presented as %(n/N); Continuous variables are presented as median (interquartile range). BAV, bicuspid aortic valve; DW-MRI, diffusion-weighted magnetic resonance imaging; TAV, tricuspid aortic valve**.**

# Supplementary Material 13

# Specific information on cerebral infarction cases.

|  | | **Group** | **BAV** | | **Total lesion volume (mm^2^)** | **Average lesion volume (mm^2^)** | **Maximum single lesion volume (mm^2^)** |  |
| --- | --- | --- | --- | --- | --- | --- | --- | --- |
| Case1 | Control | | | No | 189.84 | 94.92 | 107.578 | |
| Case2 | Control | | | Yes | 1059.375 | 132.421875 | 131.25 | |
| Case3 | Control | | | Yes | 1021.875 | 255.46875 | 571.875 | |
| Case4 | CEPD | | | No | 2104.1 | 131.507 | 511.524 | |

CEPD, cerebral embolic protection device.

# Supplementary Materials 14

# TG3 performance

|  | Control group（N=56） | CEPD group（N=62） | P-value |
| --- | --- | --- | --- |
| Successful Device Operation^a^ | NA | 100 (62/62) | NA |
| Successful Program Completion^b^ | NA | 93. 5 (58/62) | NA |
| Successful Device Retrieval^c^ | NA | 100 （62/62） | NA |

Categorical variables are presented as %(n/N);

a: The success of Device A is defined as the successful positioning and deployment of the filter at the correct anatomical location.

b: The success of the procedure is defined as the successful positioning and deployment of the filter at the correct anatomical location, deployment before TAVR, maintenance of the position during TAVR, and retrieval after the completion of TAVR.

c: The successful removal of TG3 from the body after valve implantation or after post-implantation balloon dilation of the valve.

CEPD, cerebral embolic protection device; N, number (sample size); NA, not applicable; TG3, TriGUARD 3.
